# Response to Article Published in Vol. 3. No. 1. Palliative Response to Radiotherapy in a Patient with Hypercalcemia Caused by Metastatic Adamantinoma

**DOI:** 10.1080/13577149977659

**Published:** 1999-12

**Authors:** Jeremy Whelan


*Sarcoma* (1999) 3, 197

LETTER TO THE EDITOR

### Response to article published in Vol. 3. No. 1. Palliative response to radiotherapy in a patient with hypercalcemia caused by metastatic adamantinoma

EDITOR — Lyons and colleagues report a patient with metastatic adamantinoma complicated by hypercalcemia. We too have seen such a patient in whom hypercalcemia was resistant to bisphosphonates but initially responsive to chemotherapy.

In 1980 when aged 18 the patient underwent a right above-knee amputation for adamantinoma of the right tibia. In 1993 a thoracic vertebral metastasis was excised, but recurred the following year. It was again treated by debulking and radiotherapy, the patient also undergoing removal of a single pulmonary metastasis. In 1996 extensive liver and lung metastases were noted.

At this time, serum calcium was noted to be 3.15 mmol/1 (normal range 2.2–2.6 mmol/1). Repeated infusions of pamidronate failed to improve the hypercalcemia and he was therefore treated with cisplatin 100 mg/m$^2$. There was normalisation of the serum calcium within 3 weeks, but carboplatin was subsequently substituted for cisplatin because of renal dysfunction and severe tinnitus and hearing loss.

There was no objective response in either the liver or lung disease.

The patient remained normocalcaemic for 7 months before the serum calcium rose again, to 4.02 mmol/1. There was no response to either oral or intravenous bisphosphonates or to the reintroduction of carboplatin. Oral corticosteroids were also unsuccessful and the patient died of disease 5 months later.

Clearly this is a very uncommon complication of a rare disease and indicates a poor prognosis. Lyons *et al.*, report of hypercalcemia resolving after radiation to the hemithorax is interesting. In patients who may have more widespread disease consideration should be given to the use of platinum-based chemotherapy.

Yours sincerely
Dr Jeremy Whelan
Consultant Medical Oncologist

1357-714X print/1369-1643 online/99/040197-01 © 1999 Taylor & Francis Ltd

